# Influence of Smoking Status on Fracture Healing Time: A Retrospective Cohort Analysis

**DOI:** 10.7759/cureus.99589

**Published:** 2025-12-18

**Authors:** Hamza Ahmed, Aarish Azeem, Asher Ishaq, Marium Rizwan, Farah Mazhar, Muhammad Abdulvahab

**Affiliations:** 1 Trauma and Orthopaedics, Salford Royal NHS Foundation Trust, Salford, GBR; 2 Neurosurgery, Salford Royal NHS Foundation Trust, Salford, GBR; 3 General Practice, Salford Royal NHS Foundation Trust, Salford, GBR

**Keywords:** bone metabolism, cohort study, fracture healing, impact of smoking, smoking

## Abstract

Background: Smoking is known to impair bone metabolism, yet the extent to which current, former, and never smokers differ in fracture healing outcomes remains unclear.

Objective: To evaluate the association between smoking status and radiographic time to union in adults with long-bone fractures.

Methods: This retrospective cohort included adults treated for long-bone fractures at a UK major trauma centre (2022-2025). Smoking status (current, former with at least one year of abstinence, never) was extracted from clinical records. Radiographic union was defined as bridging of at least three of four cortices; the Radiographic Union Score for Tibial (RUST) fractures supported tibial assessments. Normality and variance assumptions were assessed using Shapiro-Wilk and Levene’s tests. Differences in healing time were compared using analysis of variance (ANOVA) with appropriate post-hoc testing. Time-to-union was evaluated with Kaplan-Meier curves and multivariable Cox modelling, adjusting for age, sex, diabetes, peripheral vascular disease, open fracture, fracture site, and management. Hazard ratios (HRs) and 95% confidence intervals were reported.

Results: Of 234 patients (70 current, 70 former, 94 never smokers), healing time differed significantly among groups (20.1 ± 5.0 vs. 17.2 ± 4.0 vs. 15.3 ± 3.0 weeks; p < 0.001). Current smokers healed significantly slower than former and never smokers. Kaplan-Meier curves showed delayed union in current smokers (log-rank p < 0.001). Adjusted Cox regression demonstrated prolonged time to union among current smokers compared with never smokers (HR < 1).

Conclusion: Smoking status demonstrates a clear gradient of risk for delayed fracture healing, with current smokers experiencing the longest time to union, former smokers showing partial recovery, and never smokers healing fastest. Smoking cessation counselling should be incorporated into fracture management pathways.

## Introduction

Fracture healing is a complex biological process influenced by systemic factors, patient-related behaviours, and injury characteristics. Smoking is a recognised modifiable risk factor that impairs bone repair through vasoconstriction, hypoxia, oxidative stress, and reduced osteoblastic function [1]. These mechanisms compromise angiogenesis and callus formation, increasing the likelihood of delayed union or nonunion [2].

Although previous studies have highlighted the relationship between smoking and impaired fracture healing [3], the differential impact of current, former, and non-smokers remains insufficiently explored. Much of the existing literature treats smoking as a binary variable, overlooking the nuances between ongoing exposure, historical exposure with possible partial biological recovery, and the complete absence of tobacco use. This gap is especially relevant in modern patient populations, where smoking behaviours are shifting in response to public health initiatives, e-cigarette use, and changing societal norms. Additionally, contemporary cohorts present with varied comorbidity profiles, including diabetes, obesity, and polytrauma, that may further modulate fracture healing outcomes. Understanding how these factors interact with smoking status is essential for improved counselling, surgical planning, and postoperative expectations [4]. More granular evidence may also help guide targeted interventions such as preoperative smoking cessation, pharmacological support, or enhanced follow-up protocols.

This retrospective cohort analysis investigates the impact of smoking status on radiographic healing time among adults treated for long-bone fractures at a Major Trauma Centre in the United Kingdom between 2022 and 2025. By comparing current, former, and non-smokers, the study aims to quantify the degree to which smoking status influences union time and examine associations with complications such as delayed union or non-union. In doing so, the research seeks to provide contemporary, clinically relevant evidence that can inform both orthopaedic practice and broader public health strategies.

## Materials and methods

Study design and setting

A retrospective cohort design was justified because the exposure (smoking status) was already present before the occurrence of the outcome (fracture healing time), and complete clinical and radiographic data were available in institutional records. The cohort consisted of adults with long-bone fractures treated between 2022 and 2025 who were followed until radiographic union. The intended cause (exposure) measured was smoking status - current, former, and never smokers - while the intended effect (outcome) was time to radiographic fracture union and complications such as delayed union or nonunion.

This study received clearance and approval from the institutional research ethics committee. An informal ethical approval was obtained because of the retrospective nature, and data was given by the clinical governance team, which was then analysed. The requirement for individual informed consent was waived because it involved a retrospective review of anonymised clinical and radiographic data.

Inclusion criteria

Inclusion criteria comprised adults aged 18 years or older with radiographically confirmed long-bone fractures (femur, tibia, fibula, humerus, radius, or ulna), documented smoking status, and complete follow-up until fracture union.

Exclusion criteria

Exclusion criteria included pathological and periprosthetic fractures, patients lost to follow-up or with incomplete radiographic data, and polytrauma patients with an Injury Severity Score greater than 15.

Smoking status and exposure documentation

Smoking status was self-reported during clinical assessment and recorded in electronic medical records. Former smokers were defined as individuals abstinent from smoking for at least one year. Pack-year quantification was inconsistently available and therefore not analysed. As smoking was self-reported, misclassification bias is possible. Single-centre recruitment may limit generalisability to broader populations.

Outcome assessment

Radiographic union was defined as bridging of at least three of four cortices on orthogonal views. For tibial fractures, assessment was supported by the Radiographic Union Score for Tibial (RUST) fractures. A subset of 40 radiographs was independently reviewed by two orthopaedic clinicians to evaluate inter-observer reliability using Cohen’s kappa.

Data collection

Demographic variables, comorbidities (diabetes, peripheral vascular disease, hypertension, obesity, osteoporosis), fracture characteristics (bone, pattern, open vs. closed), and treatment modality (operative vs. non-operative) were extracted.

The primary outcome measure was radiographic healing time in weeks, while secondary outcomes included delayed union, nonunion, and related complications.

Sampling technique

A consecutive sampling approach was used to capture all eligible cases treated during the study period.

Statistical analysis

Normality was assessed using Shapiro-Wilk and Q-Q plots; Levene’s test evaluated homogeneity of variances. Continuous variables were compared using analysis of variance (ANOVA) (or non-parametric equivalents if assumptions failed). Categorical variables were analysed using χ^2^ testing.

Time to union was analysed using Kaplan-Meier survival curves with log-rank testing. A multivariable Cox proportional hazards model adjusted for age, sex, diabetes, peripheral vascular disease, open fracture status, fracture site, and treatment modality. Hazard ratios (HRs) were reported with 95% confidence intervals (CIs). A significance threshold of p < 0.05 was used.

## Results

Study population

A total of 234 patients met the inclusion criteria: 70 current smokers, 70 former smokers, and 94 never smokers. Baseline characteristics, including comorbidities and fracture patterns, are summarised in Table 1.

**Table 1:** Baseline Characteristics

Variable	Current Smokers (n = 70)	Former Smokers (n = 70)	Never Smokers (n = 94)	Chi-square
Mean age (years)	42 ± 11	45 ± 12	40 ± 10	-
Male	43 (62%)	41 (58%)	52 (55%)	0.624
Diabetes	10 (14%)	8 (12%)	8 (9%)	1.365
Peripheral vascular disease (PVD)	4 (6%)	3 (4%)	2 (2%)	1.448
Open fractures	13 (18%)	10 (14%)	9 (10%)	2.783
Operative treatment	55 (78%)	53 (75%)	68 (72%)	0.849

Healing times

Mean healing time differed significantly across smoking status groups (p < 0.001), with the longest duration observed in current smokers (20.1 ± 5.0 weeks), followed by former smokers (17.2 ± 4.0 weeks) and never smokers (15.3 ± 3.0 weeks). CIs and detailed statistical comparisons are presented in Table 2.

**Table 2:** Healing Time by Smoking Group Values were updated to include 95% confidence intervals (CIs) for all estimates.

Smoking Group	Mean Healing Time (Weeks)	95% CI
Current smokers	20.1 ± 5.0	19.0-21.2
Former smokers	17.2 ± 4.0	16.3-18.1
Never smokers	15.3 ± 3.0	14.7-15.9

Time-to-union analysis

**Figure 1:**
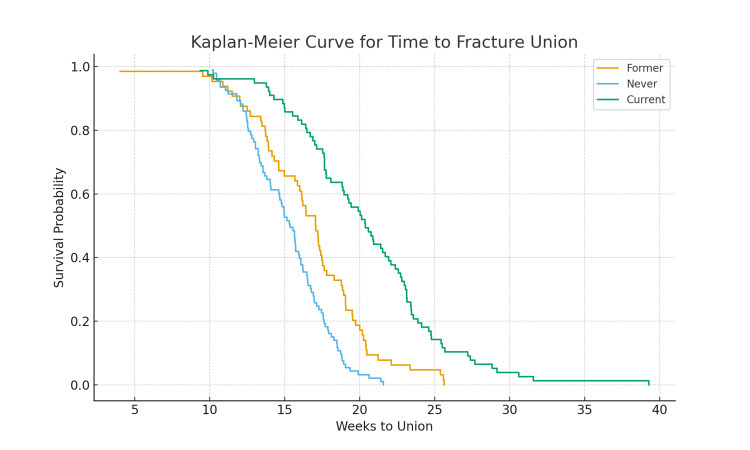
Kaplan-Meier Curve for Time to Fracture Union

Kaplan-Meier survival curves demonstrated earlier and more consistent healing among never smokers compared with former and current smokers. The log-rank test indicated significant differences among groups (p < 0.001). Figure 1 displays the survival curves.

Visual representation

**Figure 2:**
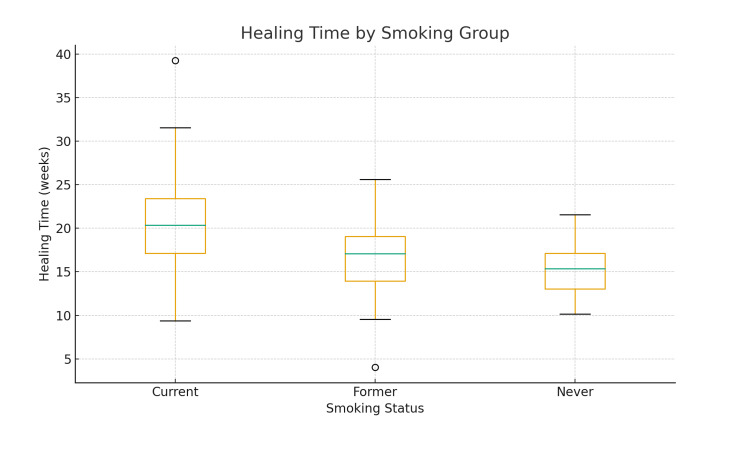
Box Plot of Healing Time Versus Smoking Status

A box plot comparing healing times among groups is shown in Figure 2, illustrating the gradient in healing duration.

Adjusted analysis

Multivariable Cox modelling showed that current smoking remained independently associated with delayed time to union after adjustment for baseline differences and injury characteristics. HRs and CIs are reported in Table 3.

**Table 3:** Multivariable Cox Proportional Hazards Model CI: confidence interval; HR: hazard ratio

Variable	Adjusted HR	95% CI	P-value
Current vs. never	0.62	0.48-0.80	<0.001
Former vs. never	0.78	0.63-0.96	0.018
Age (per year)	0.99	0.98-1.01	0.27
Male sex	1.05	0.86-1.28	0.62
Diabetes	0.84	0.65-1.10	0.21
Peripheral vascular disease	0.73	0.48-1.11	0.14
Open fracture	0.58	0.45-0.76	<0.001
Operative treatment	1.12	0.89-1.42	0.32

## Discussion

The findings of this study demonstrate a clear and clinically meaningful association between smoking status and prolonged fracture healing time among adults with long-bone fractures. Current smokers exhibited the longest healing times, followed by former smokers, while non-smokers healed the fastest. This stepwise gradient - current > former > never - indicates not only a statistical difference but also a biologically plausible dose-dependent effect, in which the extent and recency of tobacco exposure directly influence healing capacity [5].

Nicotine induces vasoconstriction and decreases peripheral perfusion [6], reducing the delivery of oxygen, nutrients, and inflammatory mediators necessary for the early stages of bone repair. This ischemic environment disrupts the inflammatory cascade, impairs angiogenesis, and limits the recruitment of osteoprogenitor cells. Additionally, carbon monoxide competitively binds to haemoglobin, reducing oxygen-carrying capacity and exacerbating tissue hypoxia [7]. Together, these mechanisms create a hostile environment for fracture healing, offering a clear explanation for the delayed union observed in current smokers [8].

Former smokers demonstrated improved healing times compared with current smokers, suggesting that cessation leads to partial restoration of vascular responsiveness, reduced systemic inflammation, and improved cellular function [9]. However, their healing remained slower than that of non-smokers, indicating that some of the detrimental effects of smoking, such as microvascular damage or impaired osteoblast activity, may persist long after cessation [10]. This highlights smoking as a risk factor with both reversible and chronic components.

The Kaplan-Meier curves further illustrate these differences by showing that current smokers reach union significantly later than both former and never smokers at nearly every time point [11]. Unlike simple comparisons of mean healing time, time-to-event analysis demonstrates the cumulative probability of union and shows a consistent delay throughout the entire healing course, reinforcing the robustness of the observed effect.

Clinically, these findings underscore the critical role of smoking cessation counselling in the management of fractures [12], particularly for patients undergoing operative fixation, where biological healing directly affects implant stability and the risk of complications. Orthopaedic surgeons should encourage cessation at the earliest opportunity - ideally [13] at initial presentation - and consider adjunct strategies such as nicotine replacement therapy or referral to structured cessation programs to reduce perioperative risk.

Importantly, this study also provides valuable insight into the intermediate risk group of former smokers. Although cessation clearly improves healing outcomes, former smokers remain at elevated risk compared with never smokers, suggesting the need for tailored follow-up and possibly more cautious postoperative monitoring [14]. Recognising this persistent risk may help clinicians better anticipate delays, optimise rehabilitation timelines, and provide more accurate prognostic information to patients.

Limitations

This study’s retrospective design limits causal inference. Smoking status was self-reported without pack-year detail, introducing potential misclassification. Radiographic union assessment, although supported by reliability testing, may maintain some subjectivity. Single-centre recruitment may reduce generalisability.

Baseline differences - including higher prevalence of diabetes, peripheral vascular disease, and open fractures among smokers - may independently delay healing. Adjusted Cox modelling demonstrated that smoking remained significantly associated with prolonged time to union even after controlling for these confounders.

## Conclusions

Smoking status is strongly associated with the time required for long-bone fracture union, demonstrating a gradient from current to former to never smokers. Current smokers experience the greatest delays, while former smokers heal faster but not to the level of never smokers. Incorporating smoking cessation counselling into acute fracture care may improve healing outcomes.
